# Nuclear Receptor Rev-erb Alpha (*Nr1d1*) Functions in Concert with *Nr2e3* to Regulate Transcriptional Networks in the Retina

**DOI:** 10.1371/journal.pone.0017494

**Published:** 2011-03-08

**Authors:** Nissa J. Mollema, Yang Yuan, Austin S. Jelcick, Andrew J. Sachs, Désirée von Alpen, Daniel Schorderet, Pascal Escher, Neena B. Haider

**Affiliations:** 1 Department of Genetics, Cell Biology and Anatomy, University of Nebraska Medical Center, Omaha, Nebraska, United States of America; 2 Department of Ophthalmology, Institute for Research in Ophthalmology, Sion, Switzerland; 3 Department of Ophthalmology, University of Lausanne, Lausanne, Switzerland; 4 Department of Ophthalmology, Ecole Polytechnique Fédérale de Lausanne, Lausanne, Switzerland; 5 Department of Ophthalmology and Visual Sciences, University of Nebraska Medical Center, Omaha, Nebraska, United States of America; University Medical Center Groningen, Netherlands

## Abstract

The majority of diseases in the retina are caused by genetic mutations affecting the development and function of photoreceptor cells. The transcriptional networks directing these processes are regulated by genes such as nuclear hormone receptors. The nuclear hormone receptor gene *Rev-erb alpha/Nr1d1* has been widely studied for its role in the circadian cycle and cell metabolism, however its role in the retina is unknown. In order to understand the role of *Rev-erb alpha/Nr1d1* in the retina, we evaluated the effects of loss of *Nr1d1* to the developing retina and its co-regulation with the photoreceptor-specific nuclear receptor gene *Nr2e3* in the developing and mature retina. Knock-down of *Nr1d1* expression in the developing retina results in pan-retinal spotting and reduced retinal function by electroretinogram. Our studies show that NR1D1 protein is co-expressed with NR2E3 in the outer neuroblastic layer of the developing mouse retina. In the adult retina, NR1D1 is expressed in the ganglion cell layer and is co-expressed with NR2E3 in the outer nuclear layer, within rods and cones. Several genes co-targeted by NR2E3 and NR1D1 were identified that include: *Nr2c1, Recoverin*, *Rgr*, *Rarres2*, *Pde8a*, and *Nupr1*. We examined the cyclic expression of *Nr1d1* and *Nr2e3* over a twenty-four hour period and observed that both nuclear receptors cycle in a similar manner. Taken together, these studies reveal a novel role for *Nr1d1*, in conjunction with its cofactor *Nr2e3*, in regulating transcriptional networks critical for photoreceptor development and function.

## Introduction

Nuclear receptors represent an evolutionarily conserved group of transcription factors that regulate genes involved in diverse functions such as homeostasis, reproduction, development, metabolism and immune response. Nuclear receptors bind to lipophilic-ligands such as steroid hormones, thyroid hormone, vitamin D and retinoids, which modulate transcriptional activity [1]–[5]. Nuclear receptors also function with co-activators or co-repressors to activate or repress the transcription of genes involved in the development and maintenance of specific cell types. In the mouse retina, nuclear receptors regulate the development and patterning of many cell types, including photoreceptor cells consisting of rods (responsible for night vision) and cones (responsible for color vision and visual acuity). The development and patterning of cone cells in mice is regulated by the nuclear receptor thyroid hormone receptor β2 (*Trβ2*, *Nr1a2b*), which controls the dorsal-to-ventral development and the terminal differentiation of medium (M)-opsin expressing cone cells [6]–[7]. Retinoid X receptor gamma (*Rxrγ*) and Retinoic acid-related orphan receptor beta (*Rorβ*) control the generation of short (S)-opsin-expressing cones (also referred to as blue cones) [8]–[9]. RORβ also induces rod photoreceptor differentiation by activating the rod-selective transcription factor Neural retina leucine zipper (*Nrl*) [10]. Rod photoreceptor function and survival is also regulated by the estrogen-related receptor β (*Errβ*) [11].

*NR2E3* (mouse *Nr2e3*) is a nuclear receptor critical for the development and maintenance of rod and cone photoreceptor cells, and loss of *NR2E3* results in disease. Mutations in human *NR2E3* cause several forms of retinal degeneration including autosomal recessive enhanced S-cone syndrome [12]–[13], Goldmann-Favre syndrome [14]–[18], clumped pigmentary retinal degeneration [14], [17], and autosomal dominant and recessive retinitis pigmentosa [19]–[21]. Loss of *NR2E3* results in a decrease in rod, long-(L) cone and M-cone function, and hypersensitivity to blue light, presumably due to an over generation of S-opsin expressing cone cells [12]. Mice lacking *Nr2e3* (*Nr2e3^rd7/rd7^, rd7*) have supernumerary blue cone cells and slow, progressive loss of rods and cones [22]–[23]. In addition, mice lacking a functional *Nr2e3* clinically display retinal spotting observable at eye opening (postnatal day 14.5), and retinal dysplasia with whorls present at postnatal day 12.5 [24]. In recent studies, NR2E3 is expressed during development in mitotic retinal progenitor cells, and functions as a suppressor of the cone generation program [24]. NR2E3 has been shown to regulate gene networks involved in photoreceptor development and function, and loss of *Nr2e3* causes mis-regulation of cone and rod genes [25]–[29].

NR2E3 functions with co-factors such as RetCoR and REV-ERB alpha (NR1D1) to regulate gene expression in the retina [25], [30]. *In vitro*, NR2E3 forms complexes with NRL, cone rod homeobox (CRX) and NR1D1 to regulate the expression of the rod phototransduction genes *Rhodopsin* and Guanine nucleotide-binding protein 1 (*Gnat1*) [25]. Recent studies also demonstrate that *Nr1d1* is up-regulated in *rd7* mice during development and in the adult retina [29]. These studies also determined that *Nr1d1* is a direct target of NR2E3 by chromatin immunoprecipitation, suggesting that these factors function in the same transcriptional network [29].

*Nr1d1* appears to have a diverse role in regulating gene networks in many tissue types and several biological processes. Additionally, activated *Nr1d1* is able to function as a monomer or a dimer, and is capable of repressing or activating gene expression, suggesting that *Nr1d1* has a diverse role in gene regulation [31]–[33]. Little is known about the role of *Nr1d1* in the mammalian retina, however its expression has been studied in a variety of tissues including skeletal muscle, liver, adipose, and eye, with its highest expression in the eye [3], [29], [34]–[38]. *Nr1d1* has been widely studied for its role in transcriptional regulation of processes such as circadian rhythm and metabolism, with heme functioning as an endogenous ligand [39]–[40]. *Nr1d1* was first identified as being encoded on the opposite strand of the thyroid hormone receptor, and was shown to bind to thyroid hormone receptor response elements, suggesting a potential role in the *Trβ2*-driven differentiation of M-opsin expressing cone cells (green cones) [6], [41]–[42]. While the role of *Nr1d1* in the retina is not well understood, recent studies indicate that it may have a crucial role in retinal development, and functions in the same transcriptional network as *Nr2e3* [29].

In this study, we determined the normal expression pattern of NR1D1 in the retina, identified co-targets of NR1D1 and NR2E3, and determined the effect of loss of *Nr1d1* in the retina. We determined that NR1D1 and NR2E3 have a similar expression profile, and that NR1D1 is expressed in retinal progenitor cells during development, and in ganglion cells and photoreceptors of the adult retina. We further identified several genes involved in phototransduction, signaling and development that are co-targeted by NR1D1 and NR2E3, in addition to genes that were individually targeted by either nuclear receptor. Acute knockdown of *Nr1d1* resulted in retinal abnormalities such as pan-retinal spotting and decreased response to light. These studies highlight an important role for *Nr1d1* in the retina and in gene regulation in concert with *Nr2e3*.

## Results

### Rev-erb alpha/NR1D1 is expressed in the developing and adult retina

**Figure 1:**
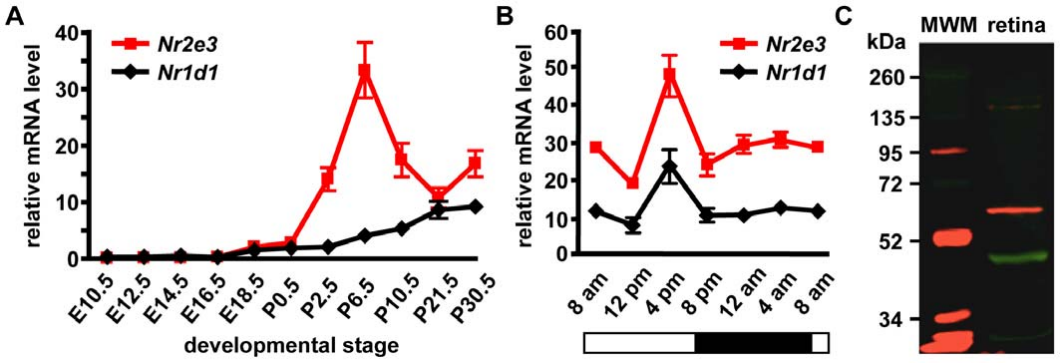
*Nr1d1* is expressed in the developing and adult retina. A: Expression of *Nr1d1* and *Nr2e3* in the developing retina. *Nr1d1* and *Nr2e3* expression begin at embryonic day (E) 18.5 with expression persisting into the adult retina. *Nr2e3* expression peaks at postnatal day (P) 6 and decreases into the adult retina, while the expression of *Nr1d1* increases into the adult retina. B: Cyclic expression of *Nr1d1* and *Nr2e3* in the adult retina. The cyclic expression pattern of *Nr1d1* and *Nr2e3* show a peak in expression of both nuclear receptors at 4 pm. Bar indicates when lights are on and off during the 12 hour light-dark cycle. Quantitative RT-PCR values corrected to fit chart scale. n = 3. C: Western blot performed with retinal lysates depicting NR1D1 protein at the expected size of 67 kDa (red). Beta actin was used as a control (green). MWM: molecular weight marker.

**Figure 2:**
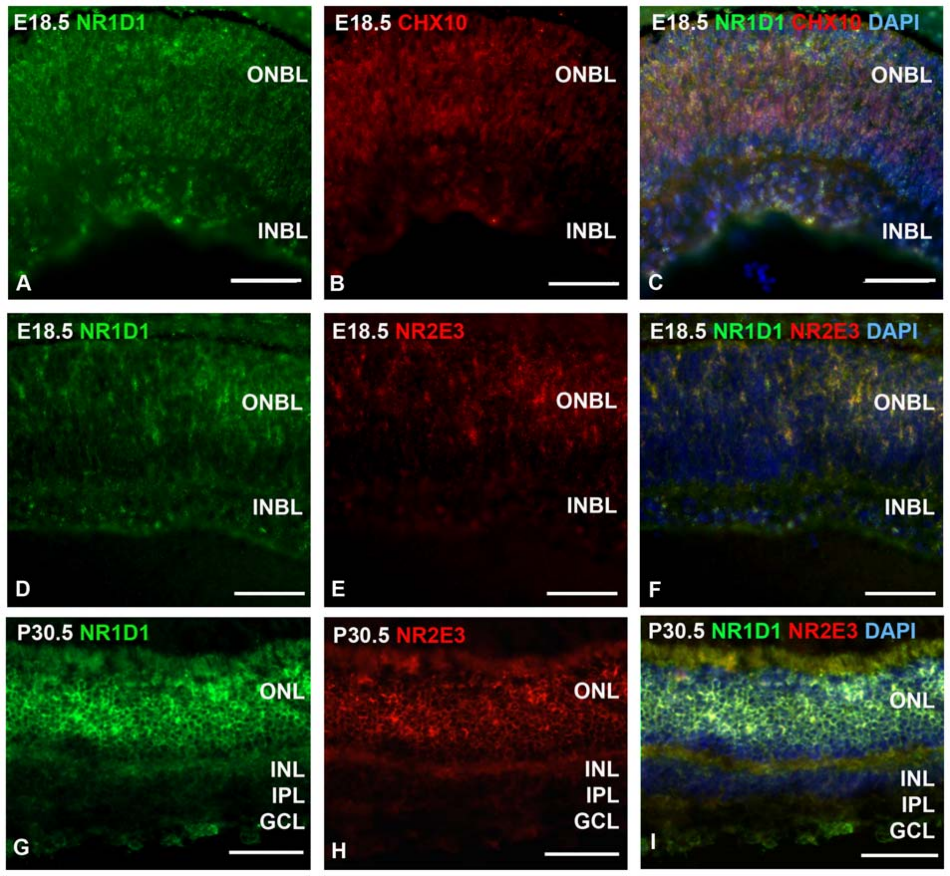
NR1D1 co-localizes with NR2E3 in the developing and adult retina. Immunohistochemical analysis shows NR1D1 localized to the outer neuroblastic layer (ONBL) and inner neuroblastic layer (INBL) in the embryonic day (E) 18.5 retina (A–C, D–F). A. NR1D1, B. CHX10, C. Merge + DAPI, D: NR1D1, E. NR2E3, F: Merge + DAPI. NR1D1 co-localized with progenitor cell markers CHX10 and NR2E3 (C, F). In the adult retina NR1D1 was expressed in the ONL, and GCL. G. NR1D1, H: NR2E3, I: Merge + DAPI. ONL: outer nuclear layer, OPL: outer plexiform layer, INL: inner nuclear layer, IPL: inner plexiform layer, GCL: ganglion cell layer. Scale bar  = 50 µm.

Quantitative RT-PCR shows that *Nr1d1* is first expressed at embryonic day 18.5 with increasing expression in the adult retina (Figure 1 A). The onset of *Nr2e3* expression is also embryonic day 18.5, with a peak in expression at postnatal day 6.5 (Figure 1 A). Because *Nr1d1* has been studied for its role in circadian rhythm, we examined the expression of *Nr1d1* and *Nr2e3* over a twenty-four hour period (8 am-8 am). Both *Nr1d1* and *Nr2e3* mRNAs showed a cycling pattern with a peak of expression at 4 pm (Figure 1 B). To determine the localization of NR1D1 protein in the mouse retina, we first performed Western blotting to confirm antibody specificity. NR1D1 protein (67 kD) was detected by Western blot in C57BL/6 (B6) postnatal day 30.5 retinal lysate (Figure 1 C). During development at embryonic day 18.5, NR1D1 protein was expressed in the outer neuroblastic layer of the retina where developing post-mitotic photoreceptors and retinal progenitors reside (Figure 2 A, B, C, D, E, F). NR1D1 co-localized with progenitor cell marker Visual system homeobox 2 (CHX10), and with NR2E3 during development (Figure 2 C, F). In the adult retina, NR1D1 was predominantly expressed in the outer nuclear layer, where rod and cone cells reside, and also localized to the ganglion cell layer (Figure 2 G).

### Loss of Rev-erb alpha/NR1D1 results in pan-retinal spotting, and a decrease in retinal response to light

**Figure 3:**
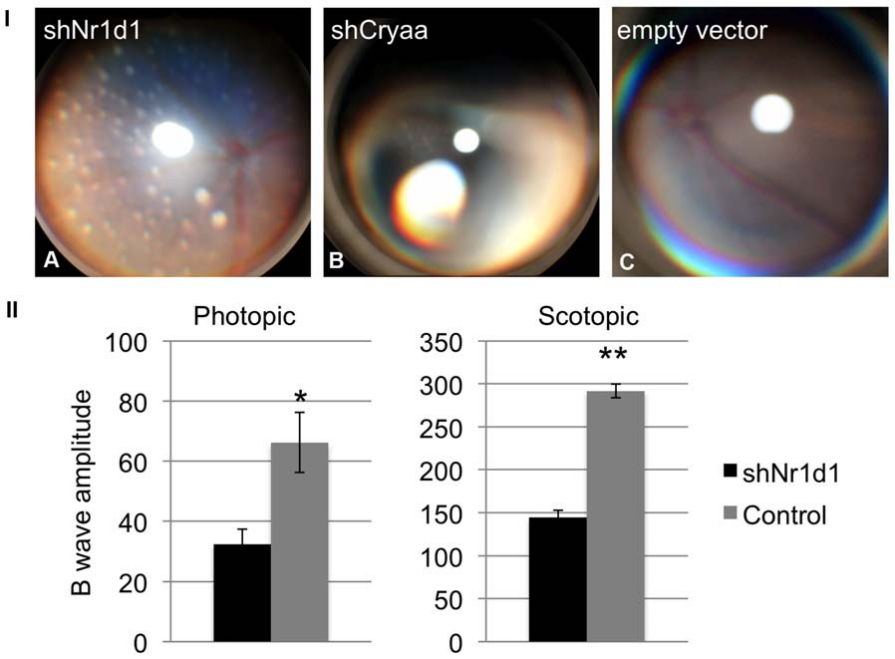
Injection with shNr1d1 results in pan-retinal spotting and reduced response to light in scotopic (dark-adapted) and photopic (light-adapted) experiments. Mice were injected at postnatal day (P) 0.5 and aged to P30.5 for clinical and functional phenotyping. I: shNr1d1 (A) produced pan-retinal spotting visible on the fundus. Injection with shCryaa (B) resulted in lens opacity. Vector only injections (C) resulted in no visible fundus phenotype. II: Graphic representation of scotopic electroretinogram testing the function of rod cells, and photopic electroretinogram testing the function of cone cells, reveled that shNr1d1 reduced the response to light by 50% compared with controls. Responses from photoreceptor cells and second order neurons were affected. shNr1d1 n = 3, B6 control n = 5. Statistical significance of a two-tailed unpaired t-test are shown as: *: p<0.05; **: p<0.01.

**Figure 4:**
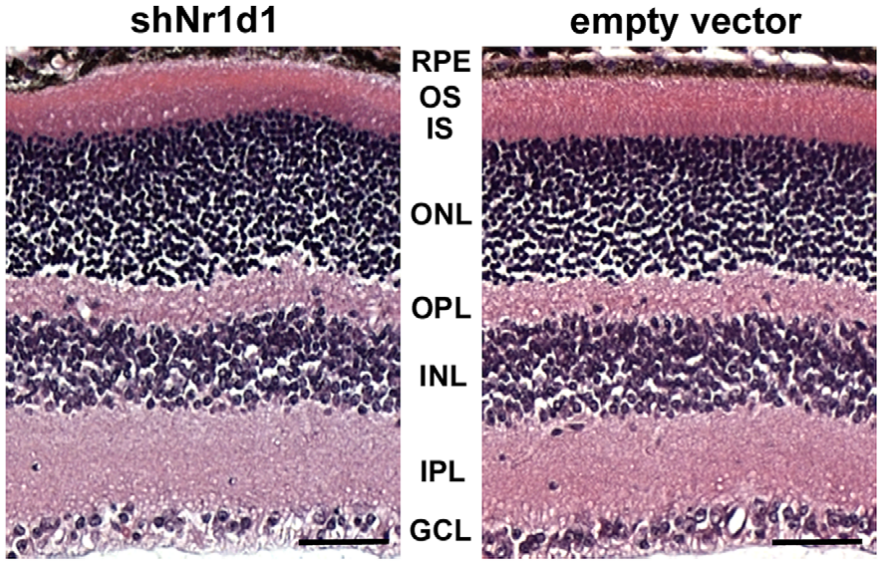
Hematoxylin and Eosin staining of shNr1d1 injected retinas does not reveal morphological abnormalities. shNr1d1 and vector only injected mice were aged to postnatal day 30.5 for analysis by Hematoxylin and Eosin staining. No gross structural abnormalities were observed in eyes injected with shNr1d1 when compared to vector only controls. RPE: retinal pigmented epithelium, OS: outer segments, IS: inner segments, ONL: outer nuclear layer, OPL: outer plexiform layer, INL: inner nuclear layer, IPL: inner plexiform layer, GCL: ganglion cell layer. n = 3. Scale bar  = 50 µm.

**Figure 5:**
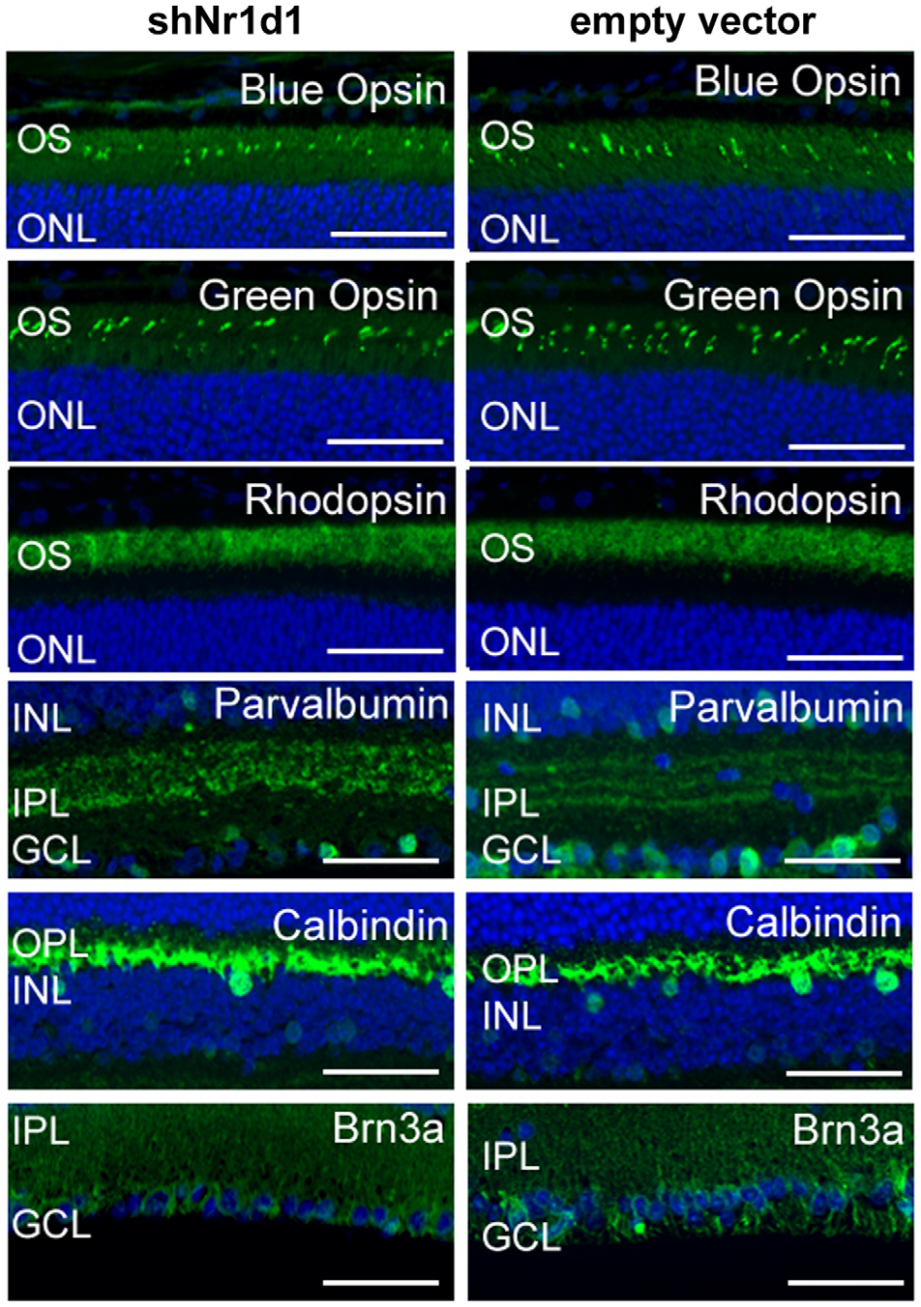
Cell type specific markers do not reveal abnormalities in retinal cell number or localization in shNr1d1 injected mice. Labeling with markers for Blue opsin (blue (S-) opsin expressing cones), Green opsin (green (M-) opsin expressing cones), Rhodopsin (Rod cells), Parvalbumin (amacrine cells), Calbindin (horizontal cells), and Brn3a (ganglion cells) did not reveal any problems with cell placement, organization or morphology within the retina when examined at postnatal day 30.5. n = 3. Scale bar  = 50 µm.

Loss of NR1D1 expression in the retina was evaluated using small hairpin (sh)RNA delivery by *in vivo* electroporation. Efficiency of gene silencing was first confirmed *in vitro* by quantitative RT-PCR (Figure S1). shRNA constructs were injected subretinally into neonate B6 eyes, and *in vivo* electroporation was used to transfect DNA into retinal cells. Mice were aged to postnatal day 30.5 for clinical, functional and histological analysis. Clinically, *Nr1d1* knockdown at postnatal day 0.5 produced pan-retinal spotting of the fundus (Figure 3 I A). Delivery of shRNA directed against crystallin Aa (*Cryaa)* resulted in lens opacity (Figure 3 I B). A normal fundus was observed in mice injected with the empty vector control (Figure 3 I C). Functionally, mice injected with shNr1d1 had 50% lower response from photoreceptor cells and second order neurons in both light-adapted (photopic- to test cone function) and dark-adapted (scotopic- to test rod function) conditions (Figure 3 II). Histological evaluation with hematoxylin and eosin staining on shNr1d1-injected mice at postnatal day 30.5 did not reveal any gross histological abnormalities (Figure 4), as are present with loss of *Nr2e3*. To identify abnormalities in gene expression, we examined shNr1d1-injected retinas with retinal cell type-specific markers at postnatal day 30.5 (Figure 5). This did not reveal any marked abnormality in abundance and distribution for any particular cell type within the retina.

### Transcriptional networks co-targeted by NR1D1 and NR2E3 are involved in phototransduction, signaling, and gene regulation

**Figure 6:**
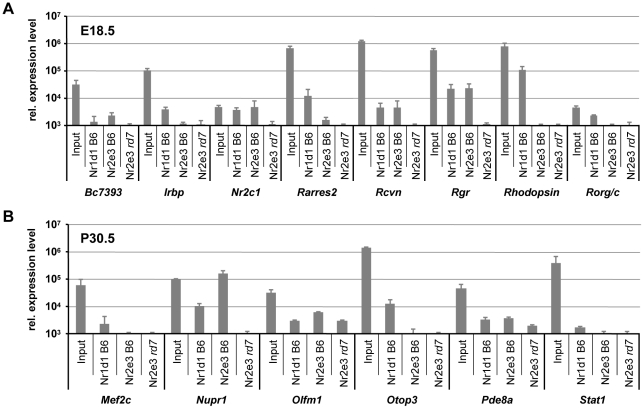
*In vivo* identification of gene targets by NR1D1 and NR2E3 in the developing and adult retina. Chromatin immunoprecipitation was conducted with Nr1d1 and Nr2e3 antibody in the developing (embryonic day 18.5) and adult (postnatal day 30.5) retina and analyzed by quantitative RT-PCR. Samples were normalized to immunoglobulin (Ig) control precipitation. X axis: expression relative to Ig. Input: positive control, Nr1d1 B6: NR1D1 ChIP in B6 retina, Nr2e3 B6: NR2E3 ChIP in B6 retina, Rd7: NR2E3 ChIP in *rd7* retina (negative control). n = 3. Data values normalized with reference to chart axis.

Microarray analysis was used to identify differentially expressed genes between B6 and *rd7* mice. The time points of embryonic day 18.5 and postnatal day 30.5 were selected as the period in which *Nr2e3* expression begins, and as an adult time point in which *Nr2e3* is expressed. Microarray results were confirmed by quantitative RT-PCR (Table S1). Recently identified target genes of NR2E3 [29], or genes identified as differentially expressed between wild type B6 and mutant *rd7* retinas by microarray analysis, were evaluated by chromatin immunoprecipitation to identify genes that are co-targeted by both NR1D1 and NR2E3. Chromatin immunoprecipitation was conducted with anti-NR1D1 and anti-NR2E3 antibodies in parallel, and samples were analyzed by quantitative RT-PCR to identify potential co-targets (Figure 6). At embryonic day 18.5, the potential regulatory regions of a total of eight out of 31 genes (∼25%) were bound by NR1D1, NR2E3 or both nuclear receptors, and at postnatal day 30.5 six out of 36 genes (∼18%) were positively targeted by one or both nuclear receptors. The genes targeted by NR1D1 and NR2E3 at embryonic day 18.5 are involved in: phototransduction (Interphotoreceptor retinoid-binding protein (*Irbp*), Retinal G Protein coupled receptor (*Rgr*), Recoverin (*Rcvn*), *Rhodopsin*); cell differentiation (RAR-related orphan receptor C (*Rorg/c*)); signaling (retinoic acid receptor responder protein 2 (*Rarres2*)); transcriptional regulation (nuclear receptor subfamily 2, group C, member 1 (*Nr2c1*)); or are novel (*Bc7393*). *Nr2c1, Rarres2, Rcvn* and *Rgr* were co-targeted by NR1D1 and NR2E3 at embryonic day 18.5. Bc7393 was targeted by NR2E3 alone, while *Irbp, Rorg/c* and *Rhodopsin* were targeted by NR1D1 alone. The genes targeted by NR1D1 and NR2E3 in the adult retina are involved in: cell growth (Nuclear protein one (*Nupr1*)), phototransduction (phosphodiesterase 8A (*Pde8a*)), signaling (signal transducer and activator of transcription 1 (*Stat1*)), transcription (myocyte enhancer factor 2C (*Mef2c*)), or novel retinal function (otopetrin 3 (*Otop3*), olfactomedin 1 (*Olfm1*)). *Nupr1* and *Pde8a* were co-targeted by NR1D1 and NR2E3; *Mef2c, Otop3* and *Stat1* were targeted by NR1D1 alone; and *Olfm1* by NR2E3 alone.

### NR1D1 and NR2E3 regulate the expression of several phototransduction genes

**Figure 7:**
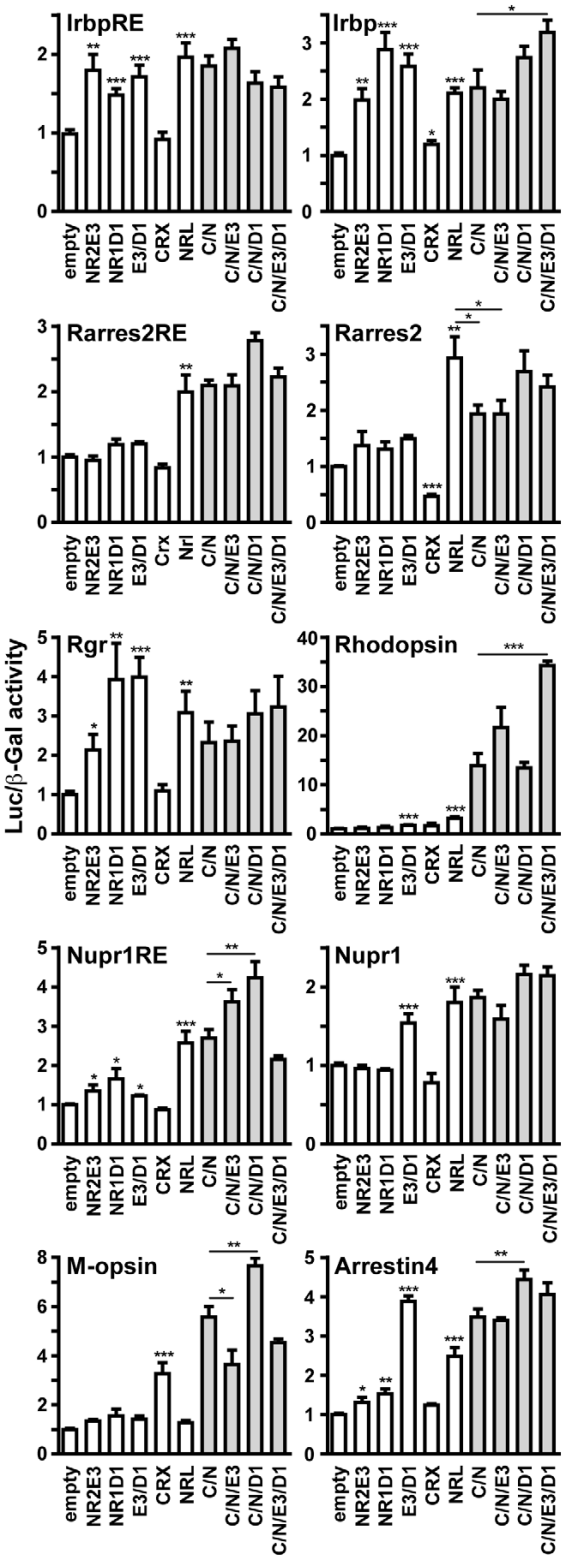
Transactivation assays on putative promoter and enhancer sequences. HEK293T cells were transiently transfected with luciferase reporter constructs containing proximal promoter or enhancer (response elements denoted as RE) regions of *Rgr, Rarres2, Nupr1, Irbp, Arrestin4* (previously called cone *arrestin 3*), *M-opsin*, and *Rhodopsin* (see Material and Methods). Transfection experiments were performed in 12-well plates, with 500 ng of luciferase reporter plasmids and 30 ng of expression vectors for NR2E3 (E3), NR1D1 (D1), CRX (C) and NRL (N) added per well. To obtain equal amounts of transfected DNA, appropriate amounts of the pcDNA3.1 plasmid (empty) were added. Luciferase activity was normalized to beta-galactosidase activity. Results are expressed as means ± SEM (n = 12–16). For samples containing both CRX and NRL, columns are colored in grey. Statistical significance of a two-tailed unpaired t-test are shown as: *: p<0.05; **: p<0.01; ***: p<0.001. If not indicated otherwise with horizontal bars, statistical significance is relative to the empty control sample. For clarity of the histograms, the significant induction of all luciferase reporter constructs in presence of both CRX and NRL is not indicated.

To determine the transcriptional activity of NR1D1 and NR2E3 on the potential regulatory regions characterized by chromatin immunoprecipitation, we cloned these sequences, as well as proximal promoter regions of the respective genes into a luciferase reporter plasmid and tested them in HEK293T cells. We tested the individual transcriptional activity of NR1D1 and NR2E3, as well as their function in a gene complex with photoreceptor-specific transcription factor CRX, and rod-specific transcription factor NRL which has previously been published for regulation of the *Rhodopsin* promoter [25]. The following genes were tested by luciferase assay: *Irbp*, *Nupr1, Rgr, Rarres2*, M-opsin, (*Mw-ops*), cone arrestin (*Arrestin4*), and, as a positive control, *Rhodopsin* (Figure 7).

With the exception of the M-opsin proximal promoter that was activated over three-fold by CRX alone, expression of all other promoter constructs was significantly induced by NRL alone. CRX alone repressed the *Rarres2* proximal promoter, and significantly decreased NRL-driven transactivation. Additionally, concurrent expression of CRX and NRL significantly increased the activity of promoter constructs. Transcriptional activity of NR1D1 and NR2E3 depended on the promoter context. For *Irbp*, the activity of the putative response site (IrbpRE) was barely modulated by the expression of transcription factors, but on the proximal promoter sequence, NR2E3 and NR1D1, alone and together, activated promoter activity by respectively 2-, 2.9- and 2.6-fold. In addition, CRX/NRL-driven transactivation was significantly increased to over 3-fold in the presence of both NR2E3 and NR1D1. The proximal promoter of *Rgr* was activated about 4-fold by NR1D1, and this activity could not be repressed by NR2E3. In presence of NRL, no other transcription factor could significantly modulate promoter activity. The proximal *Rhodopsin* promoter activity was increased by about 14-fold in presence of both CRX and NRL. This CRX/NRL-driven promoter activity was further increased to more than 20-fold in the presence of NR2E3, and even 35-fold in presence of both NR2E3 and NR1D1 The 2.6-fold CRX/NRL-driven transactivation of the *Nupr1* response element (NuprRE) was significantly increased by NR2E3 to 3.6-fold and NR1D1 to 4.2-fold. This increase in activity was suppressed when NR2E3 was added to the complex of CRX/NRL/NR1D1. A similar repressive role of NR2E3 was observed with M-opsin promoter activity. We also identified that NR1D1 and NR2E3 together were able to drive the expression of *Arrestin4* to a far greater extent than either nuclear receptor could individually, but that NR1D1 alone was able to further increase CRX/NRL-driven transactivation from 3.5- to 4.4-fold. These results illustrate that NR2E3 is able to both repress and activate gene expression and that the presence of co-factors and additional transcription factors further modulates gene expression. Furthermore, our results also indicate that NR1D1 serves a role in the activation of many photoreceptor specific genes.

## Discussion

In this study we investigated the role of NR1D1 and NR2E3 in gene regulation, and the impact of loss of *Nr1d1* on the retina. The implications of these findings aid in our understanding of retinal development and function, and identify a role for *Nr1d1* in the mammalian retina. The onset of *Nr1d1* and *Nr2e3* expression correlated with the end of cone cell generation in mice, a time period when transcriptional regulation is necessary to prevent the continued emergence of cones. While loss of *Nr2e3* resulted in continued generation of cone cells, knockdown of *Nr1d1* did not result in the over production of cones. This may indicate that genetic compensation by *Nr2e3* was able to suppress continued cone generation. Knockdown of *Nr1d1* expression did result in reduction of rod and cone function, suggesting that loss of *Nr1d1* alters the development and/or function of these cell types. Clinically, the pan-retinal spotting produced by loss of *Nr1d1* was highly similar to the pan-retinal spotting observed with loss of *Nr2e3* in mice. While loss of *Nr1d1* produced a similar clinical phenotype, histologically we did not observe gross morphological changes, as are present with loss of *Nr2e3*. While no retinal whorls were noted in our shNr1d1-injected mice, there may be additional ultra-structural abnormalities that were not detected in our studies, which could explain the observed retinal spotting and functional deficits. Additionally, our studies only examined injected mice at P30.5 and lack of *Nr1d1* may result in slow or delayed retinal degeneration. Thus, aging the mice beyond one month may result in notable histological changes, or a more severe fundus phenotype. Loss of *Nr1d1* may alter, for example, signaling pathways within photoreceptor cells, therefore limiting their capacity to fire to second order cells and decreasing retinal function. However, changes such as these may not alter the morphological integrity of the retina.

Our studies identified a physiologically important role for NR1D1 in the regulation of photoreceptor gene expression. Previous *in vitro* luciferase assay experiments had shown that the gene complex consisting of CRX/NRL/NR2E3/NR1D1 had a strong, combinatorial effect in rod-specific gene regulation that was not apparent when evaluating any single factor [25]. In our studies, we also observed combinatorial effects of these transcription factors and determined that NR1D1 and NR2E3 have dual roles in the activation and suppression of gene expression. Consistent with the previously reported suppressor activity of NR2E3 on cone-specific genes, expression of the cone-specific *M-opsin* and *Arrestin4* promoters was increased in presence of CRX/NRL/NR1D1, but NR2E3 ablated or diminished this activity [26]–[27]. For the *Irbp* proximal promoter, which had been shown previously that binding of NR2E3 was not dependent on the presence of CRX [27], NR1D1 was more efficient in driving promoter activity than NR2E3. NR1D1 also acted as a transcriptional activator on the *Rgr* proximal promoter, suggesting a more general role for NR1D1 in transcriptional activation of photoreceptor gene expression. In the heterologous cell transactivation studies, NR1D1 and NR2E3 activated the expression of the *Rhodopsin* promoter and highly potentiated CRX/NRL-driven activity. However, we did not identify NR2E3 targeting to the upstream regulatory region of *Rhodopsin* at the onset of *Nr2e3* expression *in vivo*. This could indicate that NR2E3 does not regulate the expression of *Rhodopsin* at embryonic day 18.5, but may at later time points. On the other hand, and based on previous studies [25], this also suggests that transcriptional regulation by NR2E3 is at least in part independent of binding to target promoters. Taken together, our studies demonstrate that interaction of transcription factors can have highly variable effects in the activation or suppression of gene expression depending on the regulatory region. Whereas NR1D1 preferentially activates gene expression, NR2E3 was able to both repress and activate gene expression. The presence of co-factors and additional transcription factors further modulate gene expression.

Our earlier studies with NR2E3 chromatin immunoprecipitation at postnatal day 2.5 indicated that NR2E3 targets a large subset of genes critical for photoreceptor development and function [29], suggesting gene regulation is dynamic during development. We observed that during development NR1D1 and NR2E3 co-targeted genes involved in phototransduction, signaling and transcription, indicating that both genes have the capacity to modulate genes important for retinal development. For example, during development, NR1D1 and NR2E3 co-targeted *Recoverin* (*Rcvn*), a calcium binding protein that has a critical role in the inhibition of rhodopsin kinase in rod phototransduction. The co-targeting of this gene indicates that NR1D1 and NR2E3 could suppress the expression of *Rcvn* in developing cone cells, and may explain why mice lacking *Nr2e3* develop hybrid cones that aberrantly express rod genes [43]. In the adult retina, genes co-targeted by NR1D1 and NR2E3 were involved in phototransduction and cell growth, suggesting that improper regulation of genes involved in these pathways could cause the increase in blue cone population observed in *Nr2e3^rd7/rd7^* mice.

Pathways involving phototransduction, signaling, development and cell growth are major developmental networks within the retina, and mutations in nuclear receptor genes regulating these pathways can have far reaching significant consequences. Although no human mutations in *NR1D1* have yet been reported in association with retinal degeneration, few studies have examined patients for mutations in this gene [44]. Examination of more patient cohorts may find mutations in *NR1D1* associated with retinal disease. Our studies identified a role for *Nr1d1* in gene regulation, as well as in the disease process. More studies will be needed to determine additional factors associated with the NR1D1/NR2E3 complex, as well as the dynamic gene regulation occurring in the developing and adult retina.

## Methods

### Ethics Statement

The mice used in this study were bred and maintained under standard conditions in the research vivarium at the University of Nebraska Medical Center under protocol number 04–086 approved by the Animal Care and Use Committee at the University of Nebraska Medical Center. Mice were housed in microisolator cages and provided with food and water *ad libitum*. The University of Nebraska Medical Center is in compliance with NIH policy on the use of animals in research (Animal Welfare Act P.L. 89–544, as amended by P.L. 91–679 and P.L. 94–279) and the Guide for Care and Use of Laboratory Animals, NIH Publication number 86-23. Tissues were harvested from B6.Cg-*Nr2e3^rd7/rd7^* (*rd7*) and C57BL/6J (B6) mice at embryonic day 18.5 and postnatal day 30.5. A minimum of 3 *Nr2e3^rd7/rd7^* and three B6 mice were analyzed for each time point. Mice were genotyped for the *Nr2e3^rd7/rd7^* mutation and phenotyped by indirect ophthalmoscopy for retinal spotting as previously described [13], [45].

### Western Blotting

Four eyes, enucleated from B6 mice age P30.5, were dissected and retinas were homogenized in RIPA buffer (1XTBS, 1% NP-40, 0.5% Na deoxycholate, 0.1% SDS, 0.04% Sodium azide, 1 mM PMSF). Sample concentrations were determined using the Bradford assay, and 40 μg of protein was loaded onto 4–12% gradient NuPAGE® Bis-Tris precast gels (Invitrogen). Western blot transfers were blocked in Odyssey Blocking Buffer (LI-COR) for 1 h at room temperature with rotation. Blots were incubated over night at 4°C with *Nr1d1* antibody (Sigma, mouse, 1∶1000) in 1∶1 buffer and 1X PBS. Blots were incubated with beta actin antibody (Santa Cruz Biotechnology, goat polyclonal, 1∶1000) the following morning for 1 h at room temperature. Secondary antibodies (Alexa Fluor mouse 680 from Invitrogen, IR Dye 800CW from Rockland) were incubated at 1∶10,000 dilution for 1 h in 1∶1 buffer and 1XPBS. Blots were developed using the Odyssey system.

### Immunohistochemistry

Eyes were collected, processed and immunohistochemistry was performed on 10 µm Tissue-Tek OCT embedded sections or 5 µm paraffin embedded sections from B6 and *rd7* eyes as previously described [24]. Sections were incubated with the following primary antibodies and conditions: Nr1d1 (mouse, 1∶1000, Sigma, unfixed and paraformaldehyde fixed OCT embedded), Nr2e3 (goat, 1∶100, Haider lab, unfixed OCT embedded), Chx10 (sheep, 1∶400, Chemicon, paraformaldehyde fixed OCT embedded), Blue opsin (rabbit, 1∶200, Millipore, 1∶3 methanol:acetic acid fixed, paraffin), Green opsin (rabbit, 1∶200 Millipore, 1∶3 methanol:acetic acid fixed, paraffin), Rhodopsin (mouse, 1∶500, Chemicon, 1∶3 methanol:acetic acid fixed, paraffin), Parvalbumin (rabbit, 1∶200, Abcam, 1∶3 methanol:acetic acid fixed, paraffin), Calbindin (rabbit, 1∶1000, Chemicon, 1∶3 methanol:acetic acid fixed, paraffin), and Brn3a (rabbit, 1∶400, Abcam, 1∶3 methanol:acetic acid fixed, paraffin) at 4°C overnight. Images were visualized and collected on a Zeiss Axioplan II fluorescent microscope as described previously [29].

### Cloning of mouse NR1D1 shRNA vectors

A distinct 19-nucleotide shRNA sequence against mouse *Nr1d1* was generated, using the small interference RNA selection program of the Whitehead Institute (http://jura.wi.mit.edu/bioc/siRNAext/home.php). LVm*Nr1d1*Aforward and LVm*Nr1d1*Areverse oligonucleotides for the construct were incubated at 100°C for 15 min and annealed by cooling to room temperature. Ligation was done in the presence of 100 ng of annealed oligonucleotides, and 10 ng of the pLVTHM lentiviral vector cut with *MluI* and *ClaI* (a kind gift from Dr. Didier Trono, Lausanne, Switzerland). To limit recombination of homologous plasmid sequences, bacterial transformation was done in HB101 cells. Bacterial colonies were screened by PCR using primers LVscreenfor and LVscreenrev, resulting in an amplified fragment of 271 bp in the empty pLVTHM plasmid and 325 bp in the shNr1d1 construct. Positive clones were verified by sequencing with primer LVscreenfor. An shRNA sequence directed against crystallin Aa was used as a control.

### *In vivo* electroporation

Eyes of postnatal day 0.5 B6 mice were subretinally injected with 1 µg of shNr1d1, shCryaa, or empty vector and *in vivo* electroporation was performed as described previously [46]–[47] (n>20). Plasmid DNA diluted in sterile H_2_O was pulse electroporated into the retina at 80 volts. Mice were aged to postnatal day 30.5 and phenotyped by indirect ophthalmoscopy, electroretinogram, hematoxylin and eosin staining, and immunohistochemistry as described previously [24]. Uninjected B6 mice were used as a control in electroretinogram experiments as ERGs are unaffected by plasmid DNA and procedure [48].

### Microarray analysis

RNA was isolated at E18.5 and P30.5 from B6 and *rd7* mouse retinas. Briefly, eyes were enucleated and placed in PBS on ice. Retinas were dissected using a stereo-microscope (Zeiss Stemi SV 11) and RNA was isolated by TRIzol® extraction. A total of 30 retinas were collected from B6 and *rd7* mice at similar time points during the day. Equimolar amounts of RNA isolated from ten retinas were pooled into three separate pools from both strains and time point. RNA was hybridized to Mouse 420A 2.0 (Affymetrix, Santa Clara, CA) chips by the UNMC Microarray Core Facility according to manufacturer specifications (Affymetrix, Santa Clara, CA). Data quality was assessed using the affyPLM package for the R programming language. Consistencies of expression levels were confirmed by validation across multiple redundant probe sets. Differential expression analysis was performed using the Linear Models for Microarray Analysis portion of Bioconductor. Genes found to be differentially expressed for each pair wise comparison using a FDR-adjusted p-value of 0.001 and at least a 2-fold change were combined and used to perform clustering analysis. A self-organizing map (SOM) clustering algorithm was applied to genes showing significant expression differences as judged by mean log2 intensity per strain. The gap statistic was used to estimate the optimal number of clusters. Additional analysis was performed using BRB Array Tools for Excel 2007, as well as the Stanford Statistical Analysis of Microarrays (SAM) plug-in for Excel 2007. Microarray results were confirmed by quantitative RT-PCR.

### Quantitative (real time) RT-PCR

Total RNA was isolated from embryonic day 18.5 and postnatal day 30.5 pairs of eyes from *rd7* and B6 mice using TRIzol® and samples testing positive by DNA microarray were confirmed by quantitative RT-PCR. To generate an expression profile for *Nr1d1* and *Nr2e3,* quantitative RT-PCR was performed with samples collected at 1 pm for the following time points: embryonic day (E) 10.5, E12.5, E14.5, E16.5, E18.5, postnatal day (P) 0.5 P2.5, P6.5, P10.5, P21.5 and P30.5. Gene expression studies over a twenty-four hour period were performed with retinal lysates from B6 adult (1–3 months) mice collected every four hours (12 am, 4 am, 8 am, 12 pm, 4 pm, 8 pm). Sample preparation, quantitative RT-PCR reaction and analysis were performed as described previously [29]. Primers are listed in Table S1.

### Chromatin Immunoprecipitation

Potential gene targets identified through subtractive hybridization [29] and microarray studies, were searched for classic nuclear receptor response element binding sites. Binding sites consisted of the homologous sequence “AAGTCA_(n1–4)_AAGTCA” with less than two base pair alterations from the consensus (or binding sites identified in our previous studies [29]) were selected at a maximum distance of 30 kB upstream of the ATG start site. Sixty seven genes were identified as containing testable response elements, and chromatin immunoprecipitation was performed on these putative gene targets, as described previously [29], using embryonic day 18.5 and postnatal day 30.5 retinas from B6 and *rd7* mice. Immunoprecipitation was performed overnight with 1μg of Nr1d1 antibody (Sigma), Nr2e3 antibody (Haider lab) or preimmune Ig antibody (R&D Systems). *Rd7* retinal tissue was immunoprecipitated with Nr2e3 antibody as a negative control. Quantitative RT-PCR was performed using 1μl of 1∶100 dilution (Input) and 1∶10 dilution (samples and Ig G control) using conditions described previously [49]. All sample data was normalized to Ig control. Primers are listed in Table S1.

### Luciferase assay

Mouse proximal promoter regions or upstream regulatory regions (RE) surrounding a consensus nuclear receptor response element were subcloned into *KpnI/BglII* or *SacI/BglII* sites of the pGL2-promoter luciferase reporter vector (Promega) for the following genes: *Rgr*, *Rarres2*, *Nupr1*, *Irbp*, and *Arrestin4*. Primers are listed in Table S1. *M-opsin* and *Rhodopsin* promoter constructs were obtained from Dr. Shiming Chen [27]. Full-length mouse *Nr1d1* and human *Nr2e3* cDNA were cloned into the pcDNA3.1/HisC vector (Invitrogen). All constructs were verified by sequencing on an ABI 3130xl Genetic Analyzer using the Big Dye Terminator Labeling Kit (Applied Biosystems, Carlsbad, CA). For transactivation assays, human embryonic kidney (HEK) 293T cells were grown at 37°C and 5% CO_2_ in Dulbecco's modified Eagle's medium (DMEM) (PAA Laboratories GmbH, Pasching, Austria) supplemented with 10% fetal calf serum (FCS), 100 U/ml pen and 100 µg/ml strep (Invitrogen AG, Basel, Switzerland). Cells were plated in 12-well plates (TPP, Trasadingen, Switzerland) and transfected at a confluence of 30% with the Calcium Phosphate method (ProFection®, Promega, Madison, WI). Per well, 30 ng of each of the expression vectors (pcDNA3.1/HisC-hNR2E3, pcDNA3.1/HisC-mNR1D1, pcDNA3.1/HisC-hCRX and pMT-hNRL) were used, together with 500 ng of the above-mentioned luciferase reporter constructs. As internal standard, 33 ng of plasmid CMVβ (Clontech, Mountain View, CA) encoding β-galactosidase was used. To keep the total transfected DNA quantity constant, appropriate quantities of pcDNA3.1/HisC empty vector were added in all experiments. All plasmids were prepared on NucleoBond®PC500 columns (Macherey-Nagel, Düren, Germany). Enzymatic activities were assessed with Luciferase Assay System (Promega) and standard β-Gal assay. Statistical analysis (two-tailed unpaired t-test) was performed with Prism 4.0.2 (GraphPad Software Inc., La Jolla, CA).

### Efficiency of shNr1d1

To test the efficiency of the short hairpin (sh) RNA vector in silencing mouse (m) *Nr1d1* expression (Figure S1), HEK293T cells were transiently transfected in 100-mm plates at a confluence of 30% with 2μg of pcDNA3.1-mNr1d1, a mammalian expression vector for mouse Nr1d1. Additionally, 2μg of either pLVTHM empty vector, or pLVTHM encoding a shRNA for mNr1d1 (shNr1d1) or crystallin Aa (shCryaa) were co-transfected. After 48 h of transfection, the transfection efficiency was estimated under a fluorescent microscope (Zeiss Axiovert 200) with a 20x objective, by counting the number of cells expressing the co-transfected pnlsGFP (100 ng/dish) [50]. Four regions were counted and average values used as a normalisation factor. Total RNA was then extracted with TRI Reagent® (Molecular Research Center Inc., Cincinnati, OH) according to manufacturer's instructions. Total RNA was treated with DNAse I (Applied Biosystems/Ambion, Austin, TX). Reverse transcription was performed on 500 ng of total RNA (1st strand cDNA synthesis kit for RT-PCR (AMV); Roche, Basel, Switzerland). Quantitative PCR was performed on an Mx3000p sequence analyzer (Stratagene, La Jolla, CA) using Fast Start Universal SYBR®Green Master Mix (Rox) (Roche). To assess mouse *Nr1d1* expression, primers mNr1d1for and mNr1d1rev were used. As internal standard, *Gapdh* expression was assessed with primers hGapdhfor and hGapdhrev (for primer sequences see Table S1). Statistical analysis was performed with Prism 4.0.2 (GraphPad Software Inc., La Jolla, CA).

## Supporting Information

Table S1 (XLS)

Figure S1 **Efficient silencing of mouse** ***Nr1d1*** **expression.** In human HEK293T cells transiently expressing mouse NR1D1 (mNr1d1), a short hairpin RNA (shRNA) construct targeting mNr1d1 mRNA (shNr1d1), decreased relative mRNA levels by about 30% (p = 0.0051). A control shRNA sequence directed against crystallin Aa did not interfere with mNr1d1 mRNA expression (p = 0.0838). Results correspond to three independent transfections in duplicates. Statistical significance was tested with a two-tailed paired t-test.
(DOC)
