# Supplementary figures and images for: Nuclear Receptor Rev-erb Alpha (Nr1d1) Functions in Concert with Nr2e3 to Regulate Transcriptional Networks in the Retina
Source: PLoS One. 2011 Mar 8;6(3):e17494. doi: 10.1371/journal.pone.0017494 (PMC3050883; doi:10.1371/journal.pone.0017494)

**
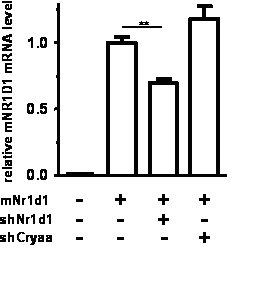
**

Supplement: Figure S1 — Efficient silencing of mouse Nr1d1 expression. In human HEK293T cells transiently expressing mouse NR1D1 (mNr1d1), a short hairpin RNA (shRNA) construct targeting mNr1d1 mRNA (shNr1d1), decreased relative mRNA levels by about 30% (p = 0.0051). A control shRNA sequence directed against crystallin Aa did not interfere with mNr1d1 mRNA expression (p = 0.0838). Results correspond to three independent transfections in duplicates. Statistical significance was tested with a two-tailed paired t-test. (DOC) [file pone.0017494.s002.doc]
